# Heterogeneous Niche Activity of *Ex-Vivo* Expanded MSCs as Factor for Variable Outcomes in Hematopoietic Recovery

**DOI:** 10.1371/journal.pone.0168036

**Published:** 2016-12-28

**Authors:** Jung-Ho Kim, Ho-Sun Lee, Hyun-Kyung Choi, Jin-A Kim, In-Sun Chu, Sun-Hee Leem, Il-Hoan Oh

**Affiliations:** 1 Catholic High-Performance Cell Therapy Center & Department of Medical Life Science, The Catholic University of Korea, Seoul, Republic of Korea; 2 Korean Bioinformation Center, Korea Research Institute of Bioscience and Biotechnology, Daejeon, Republic of Korea; 3 Department of Biological Science, Dong-A university, Busan, Republic of Korea; University of California San Diego, UNITED STATES

## Abstract

*Ex-vivo* expanded mesenchymal stromal cells (MSCs) are increasingly used for paracrine support of hematopoietic stem cell (HSC) regeneration, but inconsistent outcomes have hindered ongoing clinical trials. Here, we show that significant heterogeneity in the niche activity of MSCs is created during their culture in various serum-supplemented media. The MSCs cultured under stimulatory or non-stimulatory culture conditions exhibited differences in colony forming unit-fibroblast contents, expression levels of cross-talk molecules (Jagged-1 and CXCL-12) and their support for HSC self-renewal. Accordingly, the enhancing effects of MSCs on hematopoietic engraftment were only visible when HSCs were co-transplanted with MSCs under stimulatory conditions. Of note, these differences in MSCs and their effects on HSCs were readily reversed by switching the cultures, indicating that the difference in niche activity can be caused by distinct functional state, rather than by clonal heterogeneity. Supporting the findings, transcriptomic analysis showed distinct upstream signaling pathways such as inhibition of P53 and activation of ER-stress response gene ATF4 for MSCs under stimulatory conditions. Taken together, our study shows that the niche activity of MSCs can vary rapidly by the extrinsic cues during culture causing variable outcomes in hematopoietic recoveries, and point to the possibility that MSCs can be pre-screened for more predictable efficacy in various cell therapy trials.

## Introduction

Mesenchymal stromal cells (MSCs) are non-hematopoietic adherent cell populations derived from bone marrow (BM), adipose tissue, or placental tissue that exhibit multi-lineage differentiation potential [1, 2]. Recent studies have shown that the primary mode of action for MSCs is the paracrine support of tissue regeneration both by inhibiting apoptosis and fibrosis [3] and by stimulating the regeneration of endogenous stem cells such as hematopoietic stem cells (HSCs), neuronal stem cells, and other tissue-specific stem cells [4, 5].

In BM, the MSCs comprise both perivascular and endosteal niche [6]; a subset of mesenchymal stromal cells (MSCs) that retain colony-forming potential (CFU-F) and self-renewal ability could reconstitute both types of niches in the heterologous marrow model [7, 8]. Subsequent studies also showed that BM MSCs expressing nestin [9], leptin-receptor [10], or prx-1 [11] are enriched with CFU-F and play a major role as a niche in BM. These niche cells express various types of growth factors or ligands such as Jagged-1[12, 13] or CXCL-12 [11, 14] to regulate self-renewal [12, 15] or quiescence [16, 17] of HSCs [6].

Recently, it was shown that physiological stimuli can also alter the niche activities of MSC subpopulations and thereby induce HSCs to switch between dormant and activated states in a reversible manner [18]. Similarly, we recently showed that fine tuning the mesenchymal niche is critical for regulating the regenerative activity of HSCs [19] and that functional alterations of MSCs are related to heterogeneous clinical prognosis in hematological malignancies[20]. The niche activity of MSCs can thus exert a significant impact on the regenerative activity of HSCs.

However, MSCs are frequently prepared by ex-vivo culture with fetal bovine serum (FBS) supplements and these culture-expanded MSCs undergo functional and phenotypic changes exhibiting discrepancies from in-vivo isolated MSCs [21]. Moreover, diverse clonal heterogeneity was observed among ex-vivo expanded MSC populations with respect to their morphology, proliferation, multi-lineage differentiation and self-renewing potentials [22, 23]. Thus ex-vivo expanded MSCs are prone to heterogeneity either by selective expansion of heterogeneous clones or functional changes during culture [24].

Despite the complex heterogeneity in MSC subpopulations, ex-vivo expanded MSCs have been shown to have supportive activities for HSCs, when used in experimental models for in-vitro co-culture with murine or human HSCs [25, 26]. Similarly, studies involving co-transplantation of HSCs with MSCs have demonstrated enhancing effects on the engraftment of transplanted HSCs [27, 28]. Based on these findings, clinical trials of MSCs and HSCs co-transplantation have been pursued in multiple institutions with the goal of facilitating hematopoietic recoveries in the recipients [29].

While successive results from such clinical trials have revealed no evidence of toxicity, clinical outcomes have been highly variable irrespective of the source for HSCs used for transplantation. For example, a number of studies reported a reduced rate of graft failure with acceleration of leukocyte recovery following MSC co-transplantation [30–33]; whereas other groups reported no beneficial effect on engraftment and hematopoietic recovery [34, 35]. Thus, the factors underlying the variable outcomes in the MSC-based cell therapy have been of major interest in the field for many types of on-going clinical trials, as similarly inferred from other types of clinical trials [36–38].

In the current study, we show that significant variations in the niche activities of MSC can be created during ex-vivo expansion of MSCs to cause a variation in the outcomes of hematopoietic recoveries. Our findings thus point to the possibility that cultured MSCs can be pre-screened or optimized for more predictable outcomes in MSC-based cell therapeutic trials.

## Material and Methods

### Umbilical cord blood, MSCs and *Ex-vivo* culture

Umbilical cord blood (CB) was obtained directly from healthy pregnant woman donors under the written informed consent. The consent form and all experiments in this study had been approved by Institutional Review Board of the Catholic University of Korea (CUMC11U077). MSCs from bone marrow aspirates were also obtained under written informed consent from healthy donors under similar approval from Institutional Review Board of the Catholic University of Korea (KC13MDMS0839). For donors under age 18, the written informed consent was obtained from their parents on behalf of the children donors (MC12TNSI0120). MSC cultures were established from BM mononuclear cells and passaged in the Dulbecco’s modified Eagle’s medium (DMEM) containing 10% fetal bovine serum (FBS). During culture, different batches of FBS were purchased and tested for their effects on MSCs.

Culture of MSCs under hypoxic conditions performed by placing cells in a CO2 water-jacketed hypoxic incubator (Thermo Fisher, Heracell 150i, Waltham, MA) adjusted to 1% O_2_ as previously described [39]. To screen FBS batches, 7 and 5 of randomly chosen batches of FBS were collected from different vendors (Gibco or Hyclone) and tested for effects on MSCs in two sets of independent screening. All FBS batches were cell culture grade, filterd (3X, 100nm filter) and free of endotoxin, virus or mycoplasma.

To compare expansion in each different culture condition, MSCs that had been maintained in each culture media were sub-cultured for at least two passages before analysis. Doubling times were calculated as t/n, with t as the duration of culture and n, the number of population doublings calculated by using the formula n = log (NH-NI)/log2 (where NI is the number of cells originally plated; NH, the number of cells harvested at the time of counting).

### Animal and *in-vivo* repopulation

Experiments were undertaken with approval from the Animal Experiment Board and the Institutional Review Board of the Catholic University of Korea. C57BL/6J-Ly 5.2 (BL6) or C57BL/6J-Pep3b-Ly5.1 (Pep3b) mice were used as recipients or donors, respectively, in congenic transplantation. Enrichment of murine bone marrow cells by 5-fluorouracil treatment (5-FU BMCs) was performed as described [40]. Murine MSCs were obtained from murine bone marrow by serial passage of adherent cells in a medium containing 10% FBS until they became negative for CD45 [41]. Transplantation of the BMCs into lethally irradiated (900 rad) congenic recipient mice was performed as previously described [41]. Co-transplantation studies were performed by either simultaneous co-injection of HSC and MSCs into mice (direct) or injection of mixture of the two that had been mixed in the same test tube 2hrs before injection (priming). The repopulation of the bone marrow was assessed by measuring the proportion of donor-derived CD45.1^+^ white blood cells (WBCs) in serial peripheral blood samples. Lineages of repopulated hematopoietic cells were analyzed by immunostaining; the anti-Mac-1/Gr-1 antibody (BD Pharmingen, San Diego, CA) and anti-B220 antibodies (BD Pharmingen) were used to identify myeloid or B-lymphoid cells, respectively.

For bone marrow repopulation, mice were sacrificed 9–12 weeks after transplantation and donor-derived cells were analyzed for repopulation levels and lineages by using antibodies against CD45 (BD Pharmingen), lineage markers (StemCell Technologies, Vancouver, BC, Canada), Sca-1-PEcy7(BD Pharmingen), c-kit-APC (eBioscience, San Diego, CA, USA) by flow cytometry analysis.

### Flow cytometry of MSC

Flow cytometry analysis of MSC surface markers was performed as described [42]. Briefly, cells were stained with monoclonal antibodies, anti-human CD73-PE, CD-34-APC, CD146-PEcy7, CD271-APC, Streptavidin-PEcy7, CD140a-PE (BD Pharmingen), SSEA4-Biotin (R&D Systems, Minneapolis, MN), CD166-FITC (Serotec, Oxford, UK) and analyzed using FACSCalibur (Becton Dickinson) and CellQuest software. To examine the expression of cross-talk molecules, MSCs were permeabilized and intracellular stained with specific antibodies against Jagged-1(28H8, Cell signaling, Danvers, MA) or CXCL-12 (79018, R&D Systems) as described [13, 43]. Relative expression levels were determined by ΔMFI, difference in mena fluorescent intensity. Osteogenic differentiation and adipogenic differentiation of MSCs were induced by each specific differentiation medium and quantified by Alizarin Red staining or lipid droplet as previously described [19]. For colony formation (CFU-F), MSCs were plated at a density of 500 cells per 100 mm dish, and after incubation for 14 days, the number of colonies containing >50 cells was counted after staining with crystal violet (Sigma) in methanol.

### RT-PCR and RQ-PCR

For RT-PCR analysis, RNA was purified from MSCs and converted into cDNA using random hexamers and SuperScript^TM^ II (Invitrogen, Carlsbad, CA, USA) and amplified using specific primers for jagged-1 (5’GTG TCT CAA CGG GGG AAC TT3’ and 5’ACA CAA GGT TTG GCC TCA CA3’) or CXCL12 (5’TCA GCC TGA GCT ACA GAT GC3’ and 5’ TCA GCC TGA GCT ACA GAT GC3’). Real-time quantitative PCR (RQ-PCR) was performed with the Roter-gene 6000 system (Corbett life science, Australia) and SYBR premix Ex taq (Takara, Japan). Relative levels of PCR products were determined after normalizing to an endogenous GAPDH control. The threshold cycle (Ct) value for each gene was normalized to the Ct value of glyceraldehyde 3 phosphate dehydrogenase(GAPDH). The relative mRNA expression was calculated by using the formula; 2^-ΔΔCt^, where ΔCt = Ct_sample_—Ct_GAPDH_ and ΔΔCt = Δ Ct_sample_ -ΔCt_reference group._

### Purification, *ex-vivo* culture and long-term culture of hematopoietic cells

CD34^+^ cells were purified from mononuclear cells of UCB by using immunomagnetic cell separation (Dynabeads; Invitrogen, https://www.thermofisher.com). Cells were cultured in DMEM supplemented with each batches of fetal bovine serum (FBS) in the presence of 100ng/ml human Flt-3 ligand (Prospec Tany, Rehovot, Israel), 100ng/ml human SCF (Prospec Tany), 40ng/ml human IL-6 (R&D Systems), 40ng/ml human IL-3 (R&D Systems) and 40ng/ml human G-CSF (Prospec Tany) supplemented with 10^−6^ M hydrocortisone sodium hemisuccinate (Sigma). For co-culture, MSCs were irradiated (1500 cGy) before use and co-cultured with CD34^+^ cells in similar medium conditions for 5 days as described [13]. For colony forming assay of hematopoietic progenitors, hematopoietic cells were plated for 14 days in semi-solid methylcellulose media (MethoCult; StemCell Technologies) containing cytokines, and analyzed for colony numbers and lineages as described[13]. For long-term culture-initiating cell (LTC-IC) analysis, CD34^+^ cells were co-cultured with normal MSCs for 5 days, transferred to a 6-week long-term culture, and subjected to a colony-forming assay in semi-solid medium.

### Microarray analysis

RNA extracts were linearly amplified and hybridized to an oligonucleotide DNA microarray as described previously [44]. Briefly, double-stranded DNA template was amplified by a modified Eberwine method of the T7 RNA polymerase-based linear amplification protocol using the T7 MEGAscript kit (Ambion, Austin, TX). Biotin-labelled cRNA samples were hybridized to the Illumina Human HT-12_V4-BeadChip (48 K) (Illumina, Inc., San Diego, CA). Arrays were scanned, the array data processing and analysis were performed using Illumina BeadStudio software. Microarray study was performed by the Shared Research Equipment Assistance Program by Korea Basic Science Institute, MEST.

The hierarchical clustering was carried out using the Pearson's correlation coefficient as a distance measure with the average linkage option. The Gene Ontology (GO) program (http://david.abcc.ncifcrf.gov/) was then used to categorize genes in functional subgroups and gene set enrichment analysis was performed (GSEA) (PMID: 3999927056). In GSEA, Kolmogorov-Smirnov statistic was used to calculate the significance level of enrichment for a gene set toward the up-regulation in MSCs in stimulatory conditions versus MSCs in non-stimulatory condition. To identify candidate upstream regulators that can cause the differential transcriptomic changes in MSCs, Ingenuity Pathway Analysis (IPA, Ingenuity Systems, www.ingenuity.com) was performed.

### Statistical analysis

To determine the significance of transcriptome levels, two sample t-test was used to identify significant (p<0.01) differences between the MSCs. The overlap *P*-value in the upstream regulators, estimated by Fisher’s exact test, was used to measure a statistically significant overlap between the genes in a data set and the genes controlled by a regulator. The activation *Z*-score was estimated using signal intensities of each regulator’s targets in a data set to examine whether a potential upstream regulator is activated (positive score) or inhibited (negative score) during the pathway analysis.

## Results

### Screening MSCs cultured in different serum conditions for heterogeneous CFU-F content

**Fig 1:**
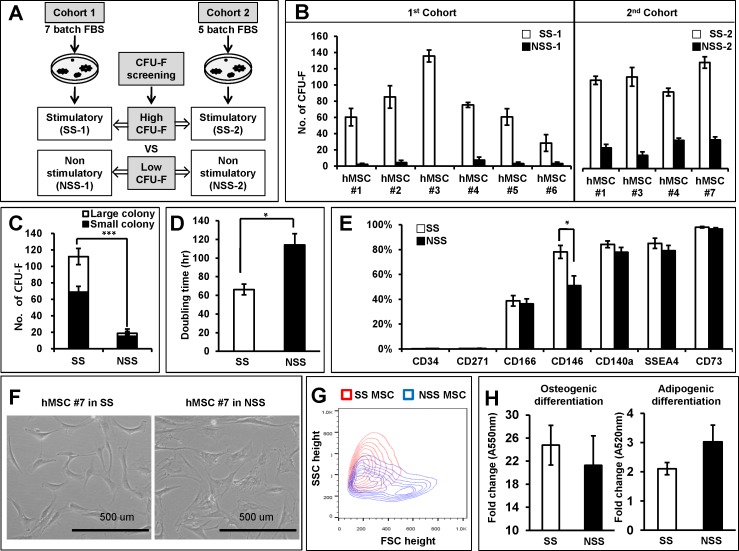
Screening of serum-supplemented culture conditions for heterogeneity of cultured MSCs. (A) Schematic illustration of experimental strategy. Culture media supplemented with multiple batches of fetal bovine serum (FBS) were screened for effects on the frequency of colony forming unit-fibroblast (CFU-F) in the cultured MSCs. Two independent cohorts each screening 7 or 5 batches of serum were tested; pairs of serum batches that can give rise to high (stimulatory serum; SS-1 or SS-2) or low (non-stimulatory serum; NSS-1 or NSS-2) content of CFU-F were identified for further analysis of their differential effects on MSCs. (B,C) Comparisons of the effects of stimulatory and non-stimulatory serum batches on the CFU-F frequencies of cultured MSCs derived from multiple donors. hMSCs derived from each independent donor (MSC#1 to MSC#7) were cultured (2 passages) and plated in stimulatory or non-stimulatory culture conditions for CFU-F during two independent cohorts of screening. Shown are the mean number of CFU-Fs (>50 cells) derived from 500 cultured MSCs after 14 days of plating with error bar representing SEM (n = 6 for each MSC line for cohort1, n = 3 for each MSC line for cohort 2) (B). Differential count of CFU-F by colony size are shown from the experiments (Large colony: >4mm, small colony: <4mm) (C). (D): Comparison of doubling times for hMSCs during culture under each serum-supplemented culture condition. (E) Effects on the surface phenotype of cultured MSCs. MSCs cultured under stimulatory or no-stimulatory conditions were compared for expression of each indicated surface marker by flow cytometry. Mean % positive cells ± SEM are shown (n = 3 for each MSC line, 3 experiments). (F) Comparisons for morphology of MSCs by light microscopy (100X). Scale bar = 500 **μ**m. (G) Comparisons for physical properties of MSCs determined by flow cytometry. MSCs under stimulatory (red) and non-stimulatory (blue) conditions were compared for forward scatter (FSC) and side scatter (SSC) in flow cytometry. Representative profiles are shown. (H) Effects on the osteogenic and adipogenic differentiation of MSCs. MSCs cultured under each conditions were subjected to osteogenic (14days) and adipogenic differentiation (16 days), and quantified by alizarin red (osteogenic) or Oil Red (adipogenic) staining, respectively, followed by spectrophotometry analysis for absorbance at each indicated wave length.

We hypothesized that heterogeneity in the stem cell-supporting activity of MSCs can be created during the ex-vivo expansion of MSCs due to differences in culture conditions. In particular, given frequent use of fetal bovine serum (FBS) to supplement the culture medium, we examined the effects of serum supplements on the functional heterogeneity of MSCs. To identify heterogeneity related to the niche activity of MSCs, we chose the frequency of CFU-F as an initial parameter for heterogeneity of cultured MSCs, based on the observations that MSCs subpopulations enriched with colony forming unit-fibroblast (CFU-F) serve as niche cells in bone marrow (BM) [9–11]. From two cohorts of screening for multiple batches of fetal bovine serum (FBS), we identified two pairs of serum batches that can give rise to high (stimulatory) or low (non-stimulatory) numbers of CFU-F (Fig 1A). These stimulatory (SS) or non-stimulatory (NSS) sera caused reproducible differences in CFU-F contents in the MSCs independently derived from 7 normal donors (Fig 1B). These results indicate that serum batches cause variations larger than those from individual donor source. When analyzed for separate effects on high- or low-proliferating colonies, different batches of serum exerted similar influences on both types of colonies (Fig 1C). Similarly, MSCs under stimulatory serum condition exhibited higher proliferating activities as evidenced by doubling times shorter than those from non-stimulating conditions (Fig 1D). When compared for surface phenotype, the MSCs under stimulatory condition exhibited a higher percentage of CD146^+^ cells than under non-stimulatory conditions, but comparable for CD34, CD271, CD140a, SSEA4 or CD73 (Fig 1E). In addition, MSCs under stimulatory condition exhibited more spindle shapes in light microscopy, whereas MSCs under non-stimulatory conditions exhibited more flattened shapes (Fig 1F) and exhibited distinct profiles in the physical properties as assessed by forward/side scatter in flow cytometry (Fig 1G and S1A Fig). However, no significant difference was seen in the osteogenic or adipogenic differentiation of MSCs under stimulatory versus non-stimulatory culture conditions (Fig 1H).

### Distinct niche activity of MSCs to cause variations in hematological recovery

**Fig 2:**
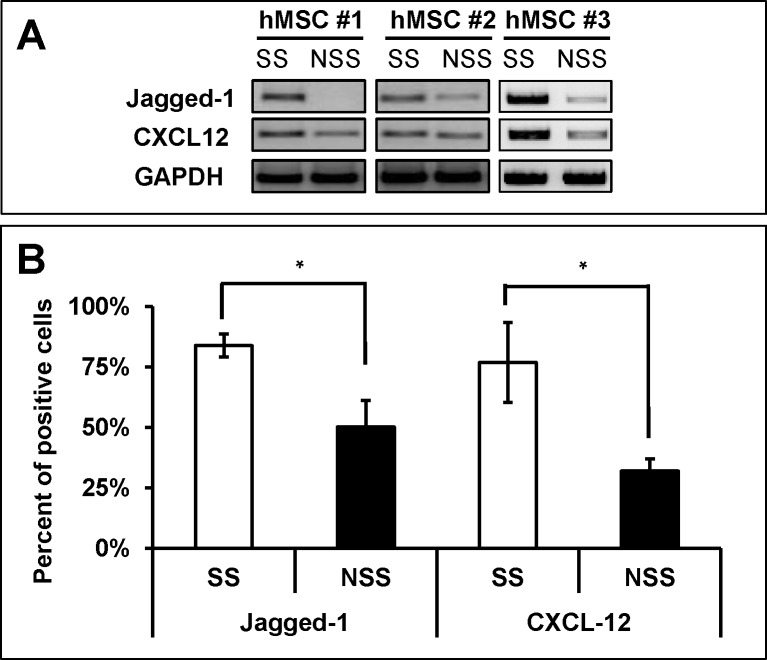
Effects of culture conditions on the expression of jagged-1 and CXCL-12 in hMSC. (A) RT-PCR analysis of expression levels for Jagged-1 and CXCL-12 genes in MSCs under each indicated culture condition. Representative plots are shown (B) Flowcytometric analysis of Jagged-1 and CXCL-12 proteins in each group MSCs by intracellular staining for the molecules. Shown are the mean % positive cells for indicated molecules with error bar representing SEM (2 expts, n = 4 each).

Next, we compared the niche activity of MSCs under different conditions to support hematopoietic stem cells (HSCs). We first compared the expression levels of Jagged and CXCL-12, the two cross-talk molecules known to play major roles in supporting HSCs both under in-vivo and in-vitro conditions [6, 13]. As shown in Fig 2A and 2B, each independent line of MSCs cultured under stimulatory conditions exhibited higher level expression of CXCL-12 and Jagged-1 than those with non-stimulatory serum, suggesting that these MSCs can show potential differences in their niche cross-talk with HSCs.

**Fig 3:**
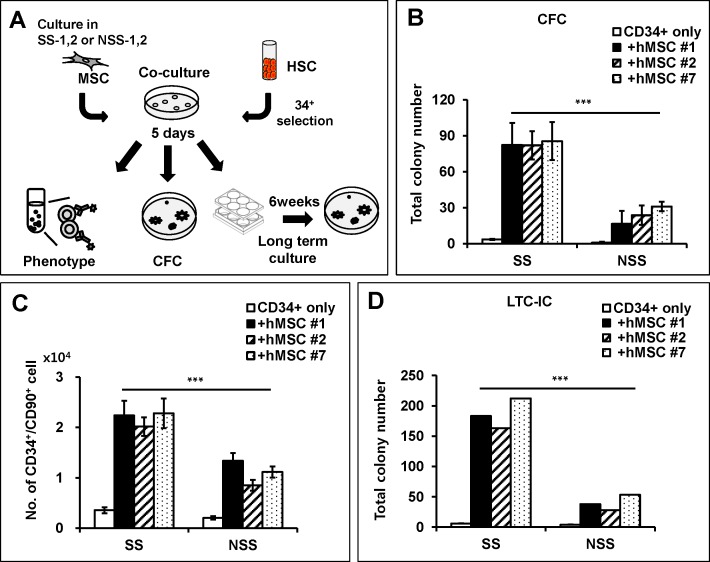
Effect of MSC culture conditions on the hematopoietic supporting activity during in-vitro co-culture. (A) Schematic illustration of the experimental scheme. CD34^+^ cells from UCB were co-cultured with hMSCs passage cultured under each serum condition (SS1,2, NSS1,2) and analyzed for their phenotype, CFC and long-term culture-initiating cells. (B) Effect of co-culture on the colony forming cells (CFC). Shown are the mean numbers ± SEM of CFCs in semi-solid medium derived from 300 input CD34^+^ cells co-cultured with each MSCs for 5 days (n = 3; ***, p < .0001). (C) CD34^+^ cells that had been co-cultured on each MSCs were analyzed for expansion of primitive hematopoietic repopulating cells (CD34^+^/CD90^+^). Total numbers of CD34^+^/90^+^ cells after co-culture were shown with SEM (n = 8; ***, p < .0001). (D) CD34^+^ cells co-cultured with each MSCs were subjected to 6 weeks’ long-term culture and analyzed for CFC content (n = 3; **, p < .001).

To examine this possibility, we compared the HSC supporting activity of each group of MSCs by co-culturing with UCB-derived human CD34^+^ cells (Fig 3A). As shown in Fig 3B, each individual donor-derived MSCs expanded under stimulatory conditions exhibited higher support of hematopoietic progenitors as reflected by higher numbers of colony forming cells (CFC) (Fig 3B). Moreover, MSCs under stimulatory conditions exhibited higher support of the primitive compartment of hematopoietic progenitors as manifested by a higher expansion of CD34^+^90^+^, the hematopoietic subpopulation with long-term SCID-repopulating activities [45] (Fig 3C) as well as higher expansion of long term culture-initiating cells (LTC-IC) detected after 6 weeks’ long-term culture [46] (Fig 3D). In contrast, such differences associated with different culture conditions were not observed when CD34^+^ cells were cultured without MSCs (Fig 3B–3D & S2 Fig). These findings are most consistent with effects arising from culture induced alterations in MSC function rather than direct effects on HSC. Taken together, these results show that MSCs can exert a distinct HSC supporting activity depending on the culture conditions with higher support of HSC self-renewal being correlated with MSC exhibiting higher frequencies of CFU-F during culture.

**Fig 4:**
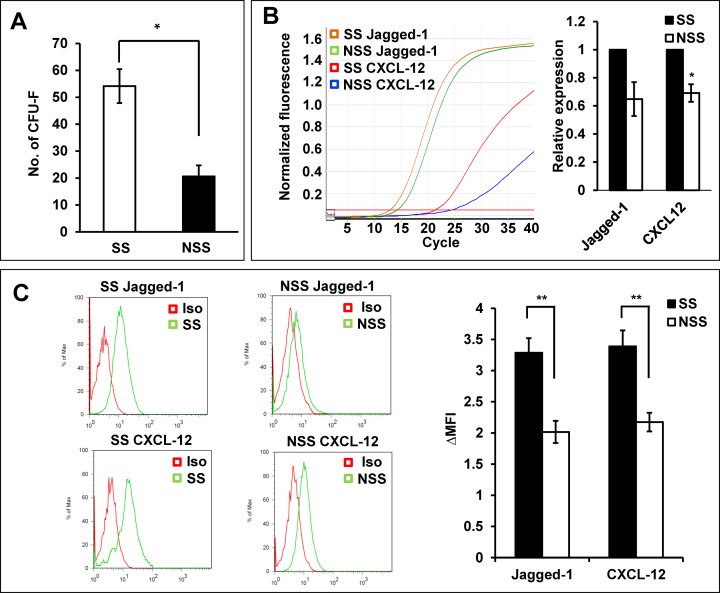
Effects of stimulatory or non-stimulatory culture conditions on murine MSCs. (A) Effects of culture conditions on murine the CFU-F in murine MSC. MSCs obtained from murine BM was cultured under stimulatory and non-stimulatory culture conditions. Shown are the mean numbers of CFU-F derived from 500 MSCs with error bar representing SEM (3 expts, n = 3 each). (B) Effects of culture conditions on the expression of cross-talk molecules. Murine MSCs cultured under each condition were compared for expression of each indicated cross-talk molecules by RQ-PCR. Shown are the representative plots (left) and quantitative comparisons of expression levels in NSS group relative to SS group as described in methods (4 expts, n = 3 each). (C) Comparisons for expression levels of cross-talk molecules by intracellular staining. Shown are the representative flow cytometry plots (left) and quantitative measurements for expression levels defined by difference in the mean fluorescent intensity relative to the isotype controls (Δ MFI) (3 expts, n = 3 each).

**Fig 5:**
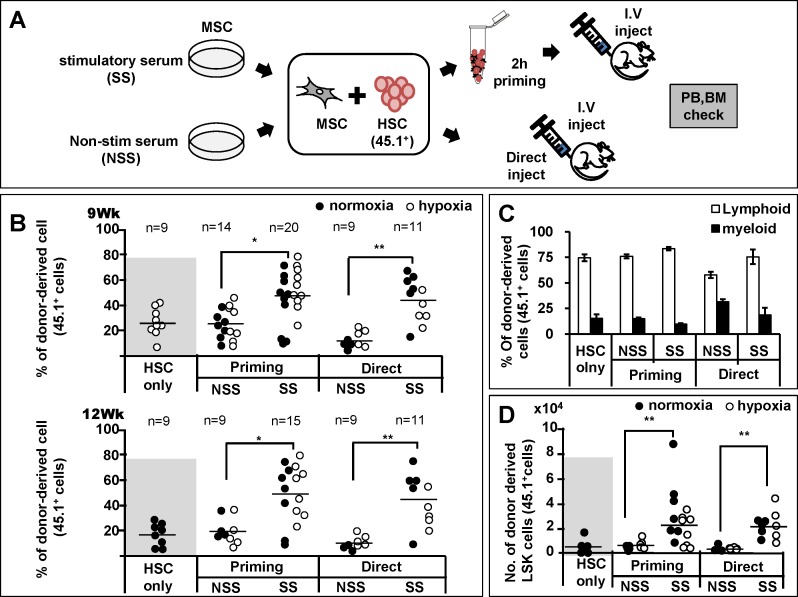
Effects of co-transplanting HSCs with MSC cultured under different conditions on the hematopoietic recovery. (A) Schematic illustration of experimental scheme. 5-FU enriched murine BM cells (2 x 10^4^) were co-transplanted with murine MSCs (2 x 10^5^) that had been expanded under stimulatory or non-stimulatory conditions into irradiated recipient mice. MSCs exposed to hypoxia (1% O_2_) for 48 hours before transplantation was also examined. Co-transplantation was either performed by simultaneous co-infusion of HSC and MSC (direct) or by injecting the mixture of HSC and MSCs that had been mixed in test tube two hours before injection (priming) along with injection of recipient origin helper cells (1 x 10^5^). (B) Effects on the peripheral engraftment of HSCs co-transplanted with MSCs. Shown are the % donor-derived peripheral blood cells in each recipient transplanted with each indicated HSCs co-transplanted with MSCs (normoxic; black, hypoxic; blank) or HSC alone without MSCs at indicated times after transplantation. Horizontal bar represents mean % engraftment (*, p<0.05, 3 expts, n = 9 for HSC only, n = 9–20 for HSCs co-transplanted with MSCs). (C) Lineage distribution of donor-derived leukocytes in peripheral blood of recipient at post-transplantation 12 weeks. The relative % of myeloid (Mac-1/Gr-1) and B-lymphoid (B220) cells are shown. (D) Comparisons for primitive hematopoietic progenitor pool size regenerated in recipient BM. Twelve weeks after transplantation in Fig 5B, the recipient mice BM were examined for numbers of donor derived LSK (Lin^-^Sca-1^+^c-kit^+^) cells for each co-transplantation group mice indicated (black: co-transplanted with normoxic MSCs, blank; co-transplanted with hypoxic MSCs). Number of donor derived LSK cells were determined by % of LSK cells in the donor-derived cells multiplied by % of donor-derived hematopoietic cells and total BM cell numbers in the recipient mice. Shown are the mean numbers ±SEM of donor-derived LSK cells in recipient (3 expts, **;p<0.01, n = 9 for HSC only, n = 9–20 for co-transplantation of HSC and MSC).

Based upon these observations, we next were interested to see if differences in MSC’s supporting activity could be a factor for variable levels of hematopoietic recoveries after co-transplantation of MSC and HSCs, mimicking the clinical trials of MSC co-infusion. For this, we employed the congenic murine repopulation model to evaluate the kinetics of engraftment in peripheral blood over 9 to 12 weeks after transplantation, considering the limitations of xenograft models to assess human hematopoietic cell engraftment in peripheral blood due to the species specificity of cytokines in murine BM [47], as well as kinetics of early hematopoietic engraftment reaching plateau in donor-derived cells [13, 40](S3 Fig). We observed that, as for human MSCs, murine BM-MSCs exhibited a similar difference in the numbers of CFU-F (Fig 4A) and expression levels of cross-talk molecules in response to the stimulatory and non-stimulatory serum batches (Fig 4B and 4C). Subsequently, donor HSCs were transplanted into lethally irradiated recipient mice together with MSCs expanded under stimulatory or non-stimulatory culture conditions. In particular, to preclude the possibility that inter-cellular contact of a mixture of HSC and MSCs might play a role, we separately examined the effects of co-transplanting HSCs and MSCs together after a two hour mixture period (priming) and in simultaneous injections into recipient without pre-mixture process (direct) (Fig 5A). As shown in Fig 5B, co-transplantation of HSCs with MSCs from non-stimulatory conditions did not cause any enhancement of early hematopoietic engraftment compared to transplantation of HSC alone for both experimental models (priming and direct injection group). In contrast, when HSCs were co-transplanted with MSCs expanded under stimulatory conditions, a significant enhancement in the engraftment levels during early phase of hematopoietic recovery was seen over 9 to 12 weeks after transplantation, for both the direct co-infusion group as well as priming group (Fig 5B). Similarly, co-injection of MSCs exposed to hypoxia (1% O_2_) for 48 hours before transplantation resulted in similar difference in engraftment levels between the stimulatory and non-stimulatory culture conditions (Fig 5B), thus indicating that the effects are reproduced for MSCs cultured under normoxic or hypoxic condition. Of note, the enhancement of engraftment was observed without a significant shift in the lympho-myeloid lineage distribution of donor-derived cells, indicating that the increase of repopulation occurred at the level of multi-lineage repopulating cells (Fig 5C). Supporting these findings, BMs of recipients transplanted with MSCs under stimulatory conditions exhibited higher levels of donor-derived stem cells numbers as indicated by a higher number of primitive (Lin^-^Sca-1^+^c-kit^+^) cells in the repopulated BM (Fig 5D). Together, these results show that the culture induced differences in the niche activity of MSCs can cause heterogeneity in the regeneration of HSCs and hematopoietic recovery.

### Reversible switch in the niche activity of MSCs

**Fig 6:**
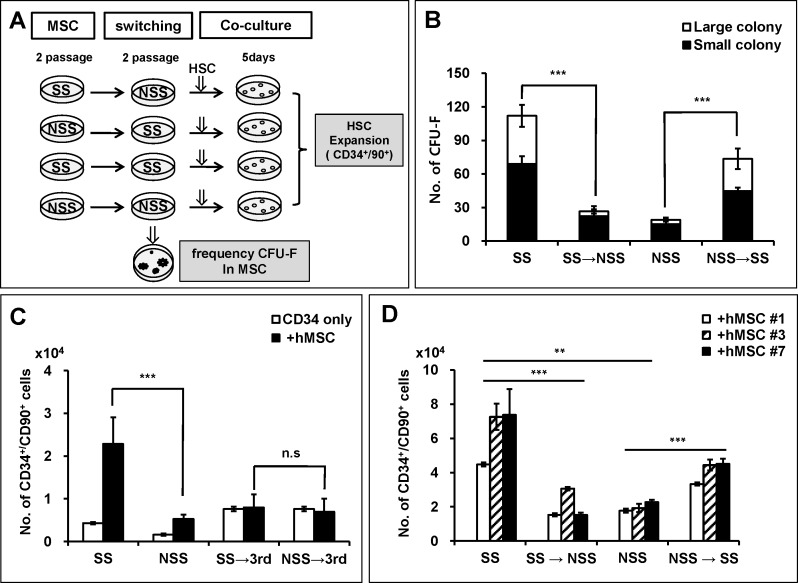
CFU-F content and niche activity of MSCs is reversed by switching culture conditions. (A) Schematic illustration of experimental scheme. MSCs that had been passage cultured in stimulatory or non-stimulatory conditions were switched to the other conditions or to 3^rd^ medium for additional 2 passages, then analyzed for CFU-F or co-cultured with HSCs for 5days. (B) Effects of switching culture on the CFU-F content of MSCs. Shown are the mean numbers of CFU-F for 500 MSC cells in each culture condition with error bar representing SEM. Large and small colonies were separately counted (n = 6 for each group). (C, D) Effects of switching culture on hematopoietic supporting activity of MSCs. MSCs that had been passaged under stimulatory or non-stimulatory condition were switched to 3^rd^ medium (C) or to the other condition (D), followed by co-culture with CD34^+^ cells for additional 5 days. Shown are the numbers of primitive hematopoietic cell population (CD34^+^CD90^+^) after co-culture under each indicated switched culture conditions with error bars representing SEM (n = 6; ***, p < .0001).

Having observed the difference in HSC supporting activities of MSCs as a function of culture conditions, we next set out to characterize the basis for the observed differences in MSC activity. We first examined whether such differences of MSCs could be ascribed to selective expansion of distinct MSC subsets leading to clonal heterogeneity between the two conditions. For this, we performed a switching culture of MSCs between different culture conditions and examined their influence on MSC function (Fig 6A). MSCs initially grown under stimulatory conditions showed a dramatic decrease of CFU-F frequencies when switched to non-stimulatory conditions. In contrast, the opposite effect was seen upon switching MSCs from non-stimulatory to stimulatory conditions as shown by recovery of their CFU-F numbers including a major difference in the number of highly proliferating (large) colonies (Fig 6B).

Moreover, when examined for the effect of switching culture conditions on MSC’s supporting activity for HSCs, the difference of stimulatory or non-stimulatory conditions on the expansion of primitive hematopoietic population (CD34^+^90^+^) was abrogated when switched into a 3^rd^ medium (Fig 6C). Similarly, stimulatory or non-stimulatory effects of each culture conditions on the expansion of CD34^+^90^+^ were reversed when the cultures were switched to the other conditions (Fig 6D), indicating that the niche activity of MSCs is concomitantly switched by the changes in culture conditions. Together, these results show that the distinction in the niche activity of MSCs most likely derives from a difference in the functional state of MSCs induced by extrinsic signals from culture conditions, rather than clonal heterogeneity caused by selective growth of distinct subsets during the culture expansion.

### Signals regulating the distinctive features of MSCs

**Fig 7:**
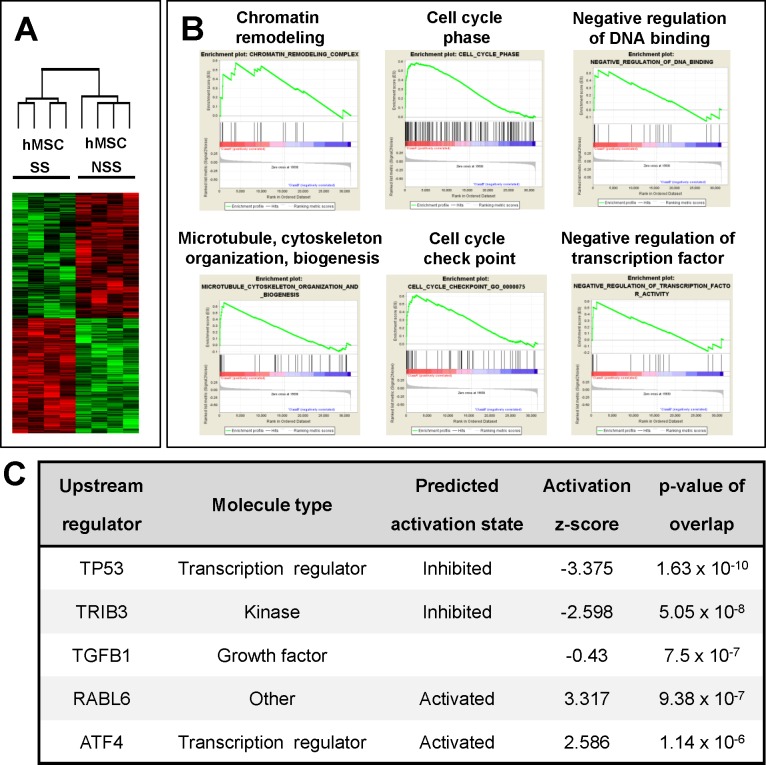
Distinct signaling pathways of the MSCs under stimulatory and non-stimulatory conditions. Three independent lines of hMSCs cultured under different conditions were subjected to microarray analysis for transcriptomic differences. Transcriptomes exhibiting significant difference (p<0.01) between the MSCs were subjected to gene set enrichment analysis (GSEA) and pathway analysis (Ingenuity pathway analysis; IPA). Shown are the plots of microarray (A) and representative enrichment profiles from GSEA (B). Full list of GOs are shown in S1 Table. (C) Distinct upstream signals of MSCs identified by pathway analysis. Five candidate upstream signals exhibiting significant alterations in the MSCs under stimulatory conditions relative to the MSCs under non-stimulatory conditions are shown.

Next, to further confirm that the different niche activity can be caused by a distinct functional status of MSCs, we first compared the gene expression profiles by microarray for three independent MSCs that had been cultured under stimulatory and non-stimulatory conditions. The result shows that, of 47,323 genes, 785 genes exhibited significant (p<0.01) differences in expression levels between the two MSC groups (Fig 7A). When these differentially expressed genes were analyzed for functional annotation by gene set enrichment analysis (GSEA), 15 of gene ontology (GO) categories were up-regulated and 4 GO categories were down regulated in stimulatory MSCs relative to non-stimulatory MSCs (FDR<25%). The list of GO categories and representative enrichment plots are shown in Fig 7B and S1 Table. These results indicate that the two groups of MSCs are indeed under distinctive signaling pathways for distinct biological function.

To identify potential upstream regulators that can cause the transcriptomic difference, we used pathway analysis (IPA, Ingenuity Systems, www.ingenuity.com) on the differentially expressed gene sets. From this analysis, we identified 5 upstream signaling pathways that exhibited most significant changes in the down-stream targets between the two MSCs functional groups; these changes included inhibition of p53 and tribbles pseudokinase 3 (TRIB3), activation of RAS oncogene family-like 6 (RABL6) and activating transcription factor 4 (ATF4) along with multiple changes in tumor-growth factor-ß signals (TGF-ß1) (Fig 7C) (downstream target molecules and signal pathway diagrams are shown in S2 Table & S4 Fig, respectively). These results suggest that MSCs cultured under stimulatory or non-stimulatory conditions are under distinct signaling pathways that may impose different niche activity.

## Discussion

For many hematological disorders, the suboptimal levels of engraftment or delayed engraftment pose higher risk of post-transplantation mortality such as increased risk of opportunistic infections and bleeding disorders, as reported for the clinical results of UCB transplantation [48, 49]. Thus various approaches have been tried to enhance the kinetics of engraftment, including ex-vivo expansion of HSCs [50] and the increase of the graft size by double cord transplantation [42, 51]. Of those approaches, clinical trials of co-transplanting MSCs and HSCs to enhance the hematopoietic recovery have been increasingly attempted, but results from multi-center trials reveal variable outcomes in hematopoietic recovery [29]. Of note, such variation was not limited to the trials for hematopoietic engraftment, i.e., similar variations were observed in the MSC-based trials to suppress graft vs host immune reactions and thus posing a dilemma in the on-going application of MSCs [36]. Moreover recent meta-analysis on the efficacy of MSC-based cell therapy in rigorously designed clinical studies point to the lack of significant efficacy in the patients with cerebral stoke [38] or myocardial infarction [37] exhibiting case variations in the outcomes. Thus, understanding the regulation of paracrine support/niche activity of MSCs now becomes of major importance for the application of MSCs in a diverse spectrum of clinical situations [52]. Accordingly various parameters such as patient factors or clinical conditions have been pointed to as potential factors for variations in the MSC-based cell therapy [36]. However, so far, the possibility that heterogeneity in MSC function might itself comprises a complicating factor has not been addressed since conventional MSC have been characterized largely by redundant surface phenotypes.

In our study, we speculated that considerable variations during MSC culture can result in expansion of MSCs different in their ability to support HSCs, and investigated influence of culture conditions on their niche activity. Accordingly, we first screened the effects of serum, since FBS has been frequently used as a culture supplement in the conventional preparation of MSCs. In addition, the frequencies of CFU-F were chosen as an initial parameter to assess their heterogeneity based on previous studies, where enrichment of CFU-F was observed among the subsets of MSCs serving as niche in BM [9–11]. Subsequently, we observed that the difference in CFU-F frequencies were associated with differences in their expression of niche cross-talk molecules such as Jagged-1 or CXCL-12 and differences in the support for HSC self-renewal in-vitro, indicating that the clonogenic activity (CFU-F) can be a parameter that can reflect the functional heterogeneity in the niche activity of MSCs, and, hence, serve as a guide for prescreening of culture-expanded MSCs.

Of note, these heterogeneities in the niche activity were related to the different outcomes in hematopoietic engraftment when co-transplanted with HSCs; the engraftment levels of donor-derived cells in peripheral blood and pool size of donor-derived HSCs in recipient BM were correlated with the CFU-F content of MSCs. Thus, the beneficial effects of co-transplanting MSC for hematopoietic recovery were only observed when HSCs were co-transplanted with MSCs that had been expanded under stimulatory conditions, but not with MSCs under non-stimulatory condition.

Interestingly, the high and low supporting activity of MSCs was reversible as observed in culture-switching experiments, where both the CFU-F content in MSCs and their supporting activity for HSCs were reversed by switching the culture medium supplemented with different serum batches. These findings suggest that the CFU-F content of ex-vivo cultured MSCs, unlike the inherent clonal differences among MSC subsets in BM, can be readily switched by extrinsic signals, and that it can be a barometer for niche activities for a variety of culture conditions including the xeno-free culture conditions currently being developed [53, 54].

Of note, we found that different batches of serum supplementation caused extensive variations in the CFU-F content of MSCs derived from 7 independent donors, whereas individual donor MSCs, under given culture conditions, exhibited comparable levels of CFU-F content and HSC-supporting niche activity as assessed by in-vivo repopulation or in-vitro culture (Fig 1B, Figs 2–5). These observations indicate that the magnitude of variation arising from culture conditions is larger than that arising from individual MSC donor populations, and that screening for culture conditions could have more influence than the donor selection screening. Taken together, these findings point to the significance of extrinsic signal-mediated regulation of niche activity and the value of CFU-F frequencies as a barometer of MSC functional status.

At present, however, the specific signals that can regulate the functional status of MSCs remain largely unclear. While we have observed that MSCs under each condition exhibited transcriptomic changes enriched with distinct functional categories in GSEA, the signaling cascades that can trigger these differences need to be further pursued. Of note, our initial efforts to search for potential upstream signals using pathway analysis (IPA) have revealed a number of candidate upstream signals that might be specific for MSCs under stimulatory condition such as TP53, TRIB3, TGF-ß1, RABL6 and ATF4. Many of these gene pathways are still poorly understood, and their significance for MSC functions currently remains largely unclear. Nevertheless, several studies provide insights on their potential relation to the functional characteristics of MSCs in stimulated phase. For example, the MSCs under stimulatory conditions exhibit significant inhibition of p53 and activation of RABL6 pathway. It was shown that RABL6, a RAS superfamily gene 6 encoding GTPase, plays a role to control cell cycle progression promoting G1 to S transition [55, 56] and negatively regulates tumor suppressor gene, p53 [57], consistent with the enhanced proliferative activity of MSCs under stimulatory conditions. In addition, inhibition of p53 was also shown to increase paracrine secretion of growth factors [58] and enhance responsiveness to TGF-ß signals [59]. Interestingly, the stimulatory MSCs exhibit significant activation of the ATF4 pathway, the ER stress-responsive gene [60]. It was shown that the activation of ATF4 in MSCs leads to stabilization of ß-catenin [61], and that ß-catenin accumulation in MSCs increases their support for HSC self-renewal and regenerative activities [13]. In addition, activation of ATF4 was associated with stress response stimulating various cytokine secretions such as VEGF-A or G-CSF [62–64]. Therefore, ATF4 could be involved by multiple mechanisms for paracrine support of HSCs. Consistent with this, deletion of ATF4 has been shown to lead to deterioration of HSC development and self-renewal with significant impairment of HSC supporting activity in niche cells [65]. Therefore, it is possible that activation of ATF4 signals in MSCs is related to the shift of MSCs towards more activated status reminiscent of the “alert state” of stem cells in response to injury stress [66]. Further studies are necessary to understand the significance of these signaling pathways and other molecular mechanisms involved in the regulation of niche activity in MSCs.

## Conclusion

Our study shows that difference in the niche activity of MSCs can be created during ex-vivo expansion resulting in variable outcomes in hematopoieic recovery. These differences originate from distinct functional state of MSCs induced by distinct upstream signals rather than clonal heterogeneity. Our study points to the possibility that the outcomes from the MSC-based cell therapies can become more predictable by pre-screening of MSCs to overcome current hurdles in the on-going clinical application of MSCs.

## Supporting Information

S1 Fig Representative flow cytometry profiles. (TIFF)

S2 Fig Direct effects of stimulatory and non-stimulatory culture conditions on hematopoietic progenitors. (TIFF)

S3 Fig Kinetics of hematopoietic engraftment in congenic murine model. (TIFF)

S4 Fig Plots for pathway analysis. (TIFF)

S5 Fig Effects of co-transplanting MSCs cultured under stimulatory or non-stimulatory conditions on early phase of hematopoietic engraftment in xenotransplantation model. (TIFF)

S6 Fig Comparisons for expression levels of CD147 between MSCs cultured under stimulatory and non-stimulatory conditions. (TIFF)

S1 Table List of gene ontology categories (GO) in MSC. (DOCX)

S2 Table Down-stream target molecules of candidate up-stream regulators exhibiting significant alterations in MSCs under stimulatory conditions relative to non-stimulatory conditions. (DOCX)
