# Supplementary figures and images for: Heterogeneous Niche Activity of Ex-Vivo Expanded MSCs as Factor for Variable Outcomes in Hematopoietic Recovery
Source: PLoS One. 2016 Dec 28;11(12):e0168036. doi: 10.1371/journal.pone.0168036 (PMC5193420; doi:10.1371/journal.pone.0168036)

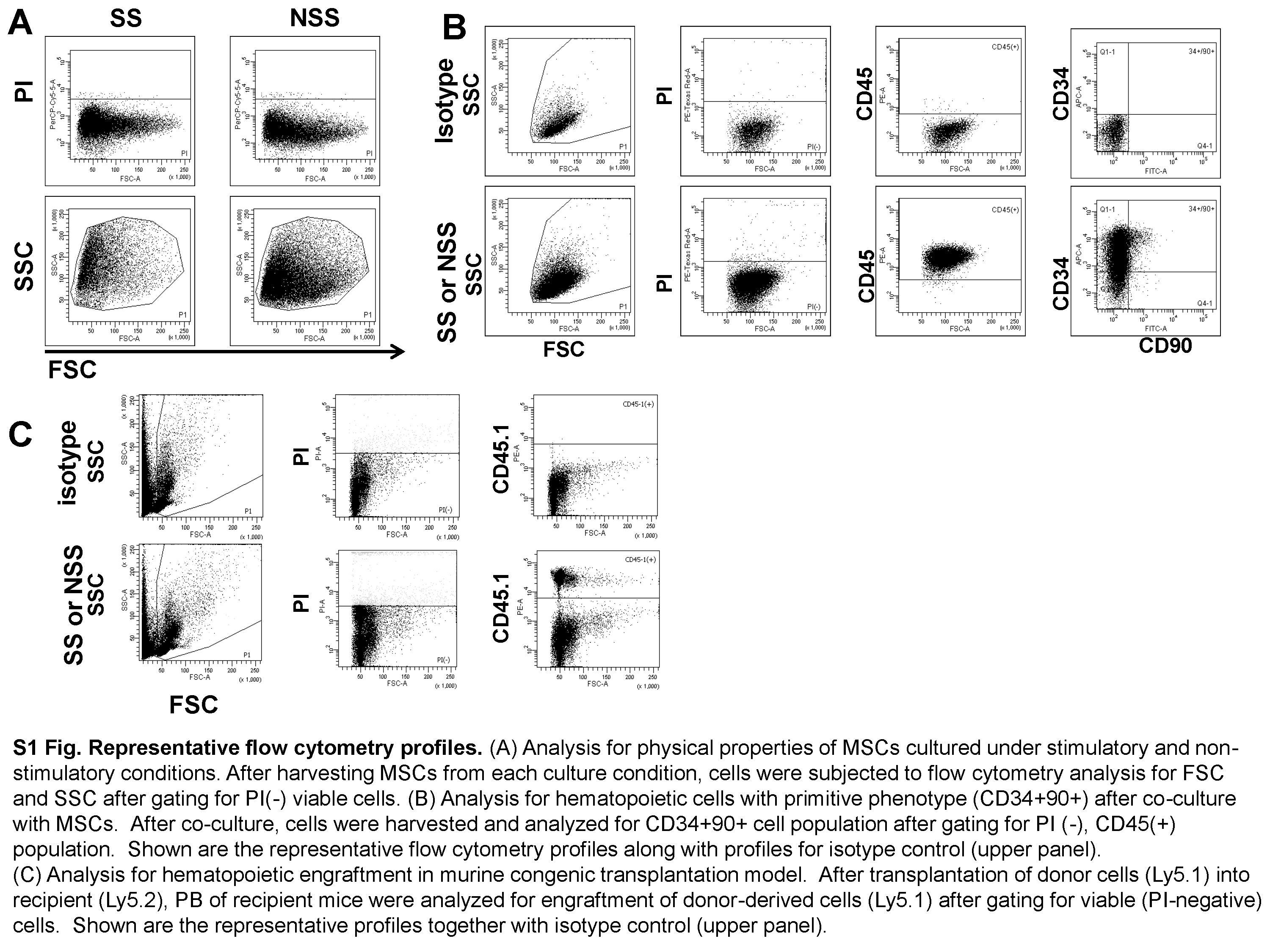

Supplement: S1 Fig — (TIFF) [file pone.0168036.s001.tiff]

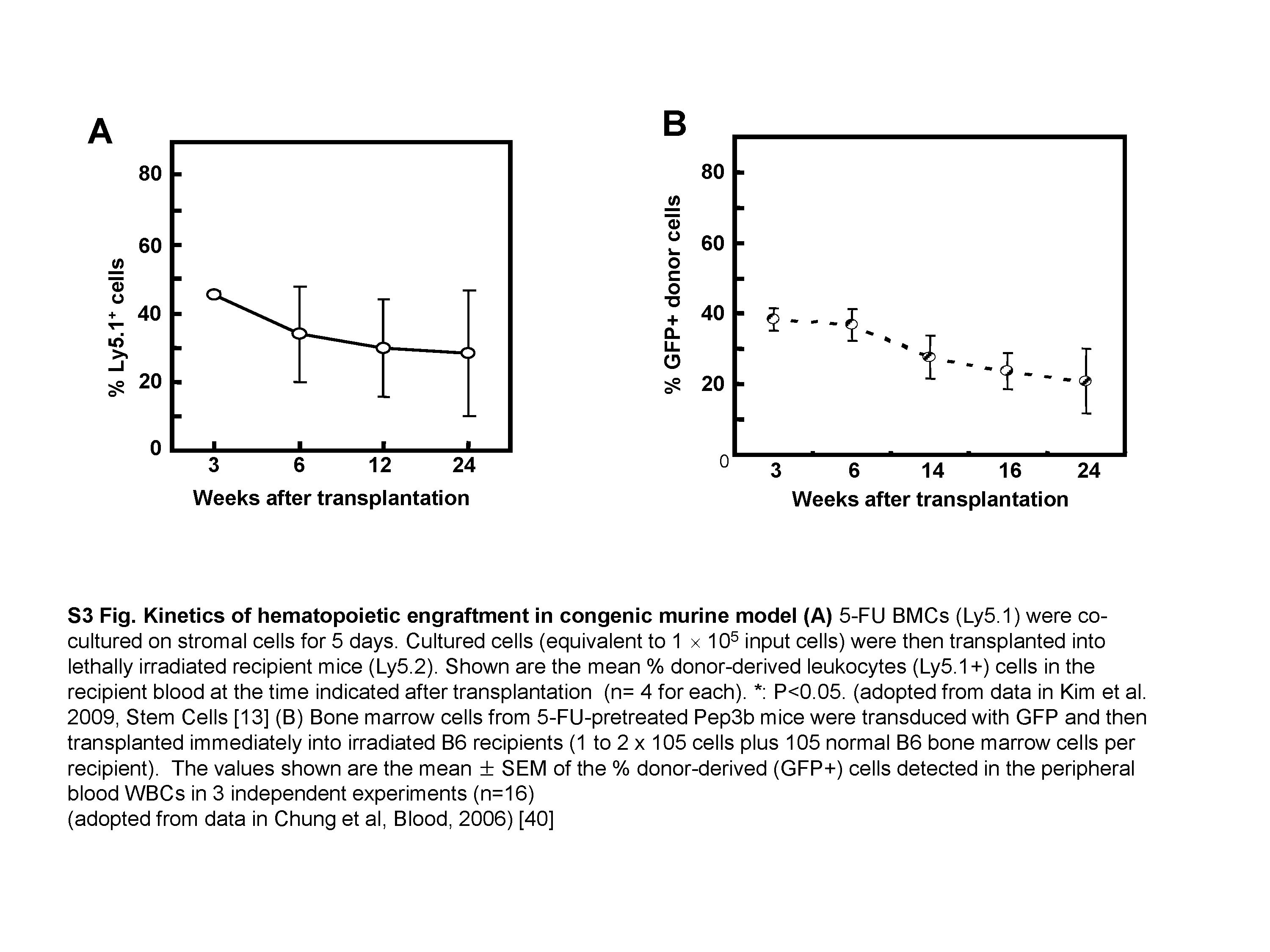

Supplement: S3 Fig — (TIFF) [file pone.0168036.s003.tiff]

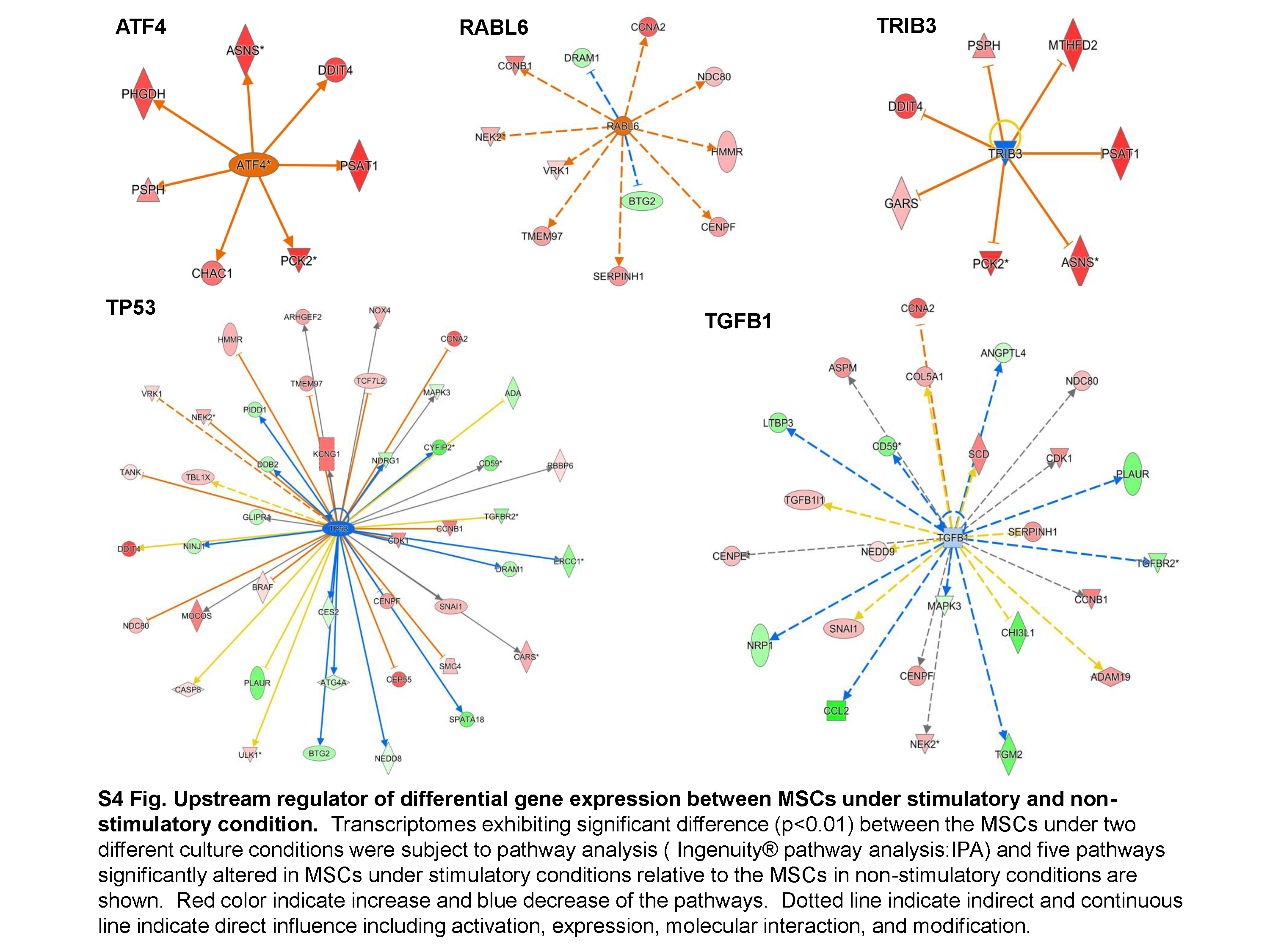

Supplement: S4 Fig — (TIFF) [file pone.0168036.s004.tiff]

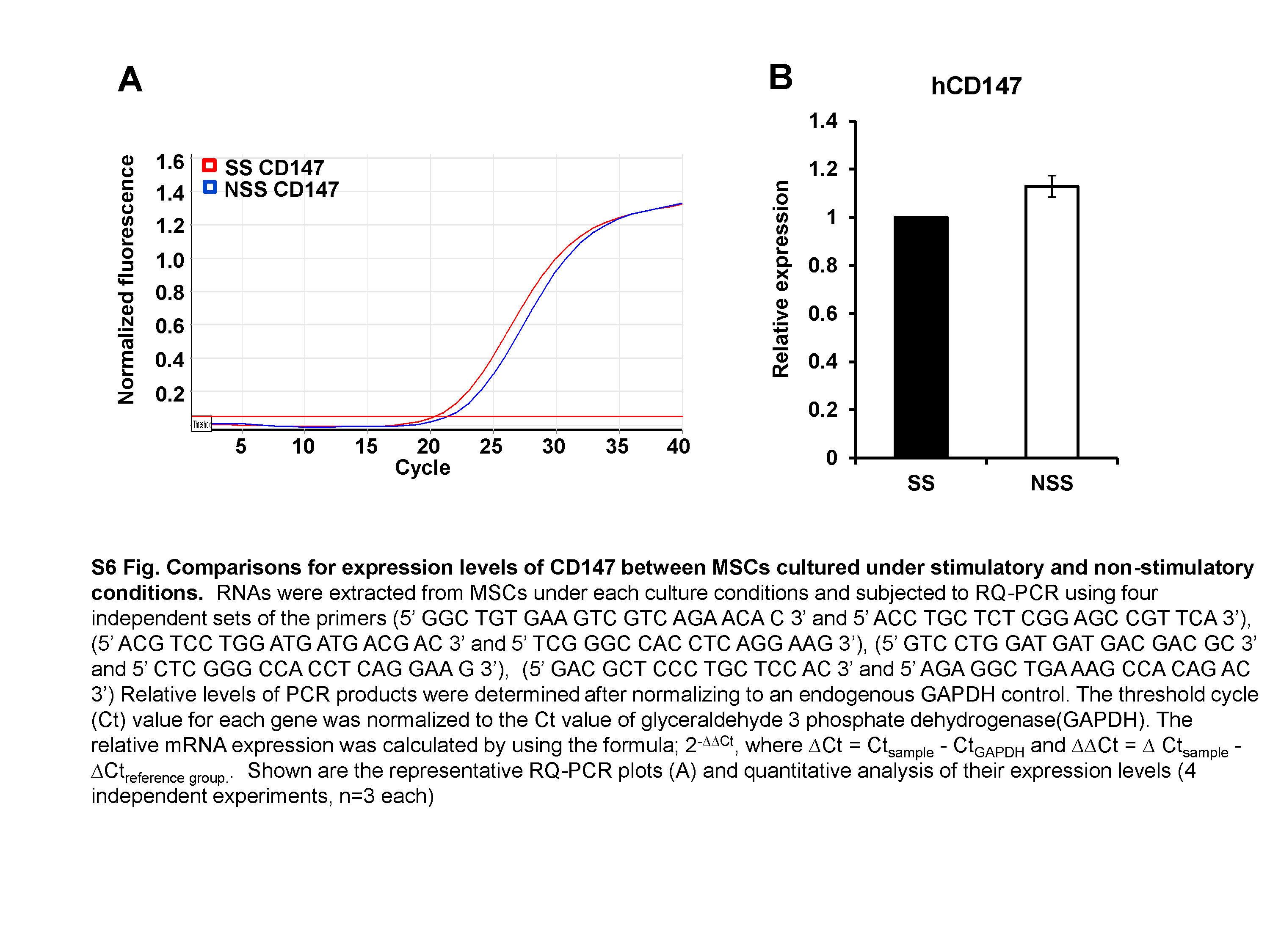

Supplement: S6 Fig — (TIFF) [file pone.0168036.s006.tiff]
