# Supplementary material for: Heterogeneous Niche Activity of Ex-Vivo Expanded MSCs as Factor for Variable Outcomes in Hematopoietic Recovery
Source: PLoS One. 2016 Dec 28;11(12):e0168036. doi: 10.1371/journal.pone.0168036 (PMC5193420; doi:10.1371/journal.pone.0168036)
Supplement: S1 Table — (DOCX) [file pone.0168036.s007.docx]

**S1 Table. (A)**

List of gene ontology categories (GO) up-regulated in MSCs under stimulatory conditions relative to MSCs under non-stimulatory conditions

| **GS** | **Size** | **ES** | **NES** | **FDR**  **p-value** | **FWER**  **p-value** |
| --- | --- | --- | --- | --- | --- |
| Nuclear pore | 31 | 0.69 | 1.8 | 0.13 | 0.06 |
| Nuclear membrane part | 42 | 0.6 | 1.71 | 0.238 | 0.269 |
| Microtubule cytoskeleton organization and biogenesis | 34 | 0.66 | 1.69 | 0.182 | 0.303 |
| Negative regulation of transcription factor activity | 15 | 0.59 | 1.68 | 0.139 | 0.303 |
| Pore complex | 36 | 0.6 | 1.65 | 0.126 | 0.347 |
| Nuclear membrane | 50 | 0.57 | 1.61 | 0.181 | 0.477 |
| Adenylate cyclase activation | 19 | 0.6 | 1.58 | 0.23 | 0.8 |
| Negative regulation of  DNA binding | 17 | 0.54 | 1.58 | 0.22 | 0.814 |
| Organelle location | 23 | 0.73 | 1.58 | 0.203 | 0.814 |
| Chromatin remodeling complex | 17 | 0.58 | 1.58 | 0.19 | 0.814 |
| Cell cycle phase | 166 | 0.59 | 1.56 | 0.221 | 0.843 |
| M phase | 110 | 0.63 | 1.55 | 0.23 | 0.856 |
| Spindle pore | 18 | 0.79 | 1.54 | 0.226 | 0.87 |
| Thyroid hormone receptor biding | 17 | 0.53 | 1.54 | 0.222 | 0.886 |
| Mitotic cell cycle | 151 | 0.58 | 1.54 | 0.21 | 0.886 |

**S1 Table. (B)**

List of gene ontology categories (GO) down-regulated in MSCs under stimulatory conditions relative to MSCs under non-stimulatory conditions

| **GS** | **Size** | **ES** | **NES** | **FDR**  **p-value** | **FWER**  **p-value** |
| --- | --- | --- | --- | --- | --- |
| Protein amino acid lipidation | 24 | 0.62 | 1.95 | 0.01 | 0 |
| Humoral immune response | 32 | 0.57 | 1.72 | 0.141 | 0.242 |
| Hydrolase activity hydrolyzing  O glycosyl compounds | 37 | 0.46 | 1.61 | 0.243 | 0.685 |
| Antioxidant activity | 17 | 0.63 | 1.61 | 0.214 | 0.685 |
