# Supplementary material for: Heterogeneous Niche Activity of Ex-Vivo Expanded MSCs as Factor for Variable Outcomes in Hematopoietic Recovery
Source: PLoS One. 2016 Dec 28;11(12):e0168036. doi: 10.1371/journal.pone.0168036 (PMC5193420; doi:10.1371/journal.pone.0168036)
Supplement: S2 Table — (DOCX) [file pone.0168036.s008.docx]

**S2 Table.**

Down-stream target molecules of candidate up-stream regulators exhibiting significant alterations in MSCs under stimulatory conditions relative to non-stimulatory conditions

| **Upstream regulator** | **Molecule type** | **p-value of**  **overlap** | **Target molecules**  **in dataset** |
| --- | --- | --- | --- |
| TP53 | Transcription regulator | 1.63 x 10^-10^ | ADA,ARHGEF2,ATG4A,BRAF,BTG2,CARS,CASP8,CCNA2,CCNB1,CD59,CDK1,CENPF,CEP55,CES2,CYFIP2,DDB2,DDIT4,DRAM1,ERCC1,GLIPR1,HMMR,KCNG1,MAPK3,MOCOS,NDC80,NDRG1,NEDD8,NEK2,NINJ1,NOX4,PIDD1,PLAUR,RBBP6,SMC4,SNAI1,SPATA18,TANK,TBL1X,TCF7L2,TGFBR2,TMEM97,ULK1,VRK1 |
| TRIB3 | Kinase | 5.05 x 10^-8^ | ASNS,DDIT4,GARS,MTHFD2,PCK2,PSAT1,PSPH |
| TGFB1 | Growth factor | 7.5 x 10^-7^ | ADAM19,ANGPTL4,ASPM,CCL2,CCNA2,CCNB1,CD59,CDK1,CENPE,CENPF,CHI3L1,COL5A1,LTBP3,MAPK3,NDC80,NEDD9,NEK2,NRP1,PLAUR,SCD,SERPINH1,SNAI1,TGFB1I1,TGFBR2,TGM2 |
| RABL6 | Other | 9.38 x 10^-7^ | BTG2,CCNA2,CCNB1,CENPF,DRAM1,HMMR,NDC80,NEK2,SERPINH1,TMEM97,VRK1 |
| ATF4 | Transcription regulator | 1.14 x 10^-6^ | ASNS,CHAC1,DDIT4,PCK2,PHGDH,PSAT1,PSPH |
